# Classifying the features of digital mental health interventions to inform the development of a patient decision aid

**DOI:** 10.1371/journal.pdig.0000752

**Published:** 2025-03-26

**Authors:** Gemma Bradley, Lucia Rehackova, Kayleigh Devereaux, Tor Alexander Bruce, Victoria Nunn, Liam Gilfellon, Scott Burrows, Alisdair Cameron, Rose Watson, Katie Rumney, Darren Flynn

**Affiliations:** 1 Department of Social Work, Education and Community Wellbeing, Faculty of Health and Life Sciences, Northumbria University, United Kingdom; 2 Department of Nursing, Midwifery and Health, Faculty of Health and Life Sciences, Northumbria University, United Kingdom; 3 Psychological Wellbeing Practitioner, Newcastle upon Tyne, United Kingdom; 4 Everyturn Mental Health, Newcastle upon Tyne, United Kingdom; 5 Patient and Public Involvement Contributor, Newcastle upon Tyne, United Kingdom; 6 Recovery College Collective, Newcastle upon Tyne, United Kingdom; 7 Strathclyde Business School, University of Strathclyde, Glasgow, United Kingdom; Iran University of Medical Sciences, IRAN, ISLAMIC REPUBLIC OF

## Abstract

Digital mental health interventions (DMHIs) are a potential scalable solution to improve access to psychological support and therapies. DMHIs vary in terms of their features such as delivery systems (Websites or Apps) and function (information, monitoring, decision support or therapy) that are sensitive to the needs and preferences of users. A decision aid is warranted to empower people to make an informed preference-based choice of DMHIs. We conducted a review of features of DMHIs to embed within a patient decision aid to support shared decision-making. DMHIs, with evidence of availability in the United Kingdom (UK) at the time of the review, were identified from interactive meetings with a multi-disciplinary steering group, an online survey and interviews with adults with lived experience of using DMHIs in the UK. Eligible DMHIs targeted users age ≥16 years with a mental health condition(s), delivered through a digital system. A previous classification system for DMHIs was extended to eight dimensions (Target population; *System; Function*; *Time*; *Facilitation*; Duration and Intensity; and Research Evidence) to guide data extraction and synthesis of findings. Twenty four DMHIs were included in the review. More than half (*n* = 13, 54%) targeted people living with low mood, anxiety or depression and were primarily delivered via systems such as Apps or websites (or both). Most DMHIs offered one-way transmission of information (*n* = 21, 88%). Ten (42%) also had two-way communication (e.g., with a healthcare provider). Eighteen (75%) had a function of therapy, with seven and five DMHIs providing monitoring and decision support functions respectively. Most DMHIs were capable of being self-guided (*n* = 18,75%). Cost and access were primarily free, with some free via referral from the UK NHS or through corporate subscription for employees (*n* = 11). Eight (33%) DMHIs had evidence of effectiveness from randomised controlled trials. Six statements were developed to elicit user preferences on features of DMHIs: Target Population; Function; Time and Facilitation; System; Cost and Access; and Research Evidence. Preference elicitation statements have been embedded into a prototype decision aid for DMHIs, which will be subjected to acceptability and usability testing.

## Author summary

Digital mental health interventions (DMHIs) such as mobile applications (Apps), internet websites and video calls are growing in use globally. DMHIs potentially help with improving access to mental health support, and earlier access to therapy than in-person services. People can access DMHIs as part or single component of mental health therapy and support, or combined with traditional in-person interventions, but making decisions from amongst the available options is complicated for individuals and healthcare professionals who support them. In this review we aimed to develop a way of classifying DMHIs based on their features to inform the design of a decision aid. With support from a healthcare professional, the decision aid would help potential users of DMHIs to choose single or multiple DMHIs that best meets their needs. We identified DMHIs that people told us they had used in the United Kingdom. We then classified each DMHI based on their features to develop a classification system. We have added this classification system to a prototype decision aid that will be tested for its usability to support shared decision-making discussions between people with mental health support needs and healthcare professionals.

## Background

In 2019, almost 1 billion people across the world were living with a mental health condition and it is estimated that depressive disorders increased by 28%, and anxiety disorders by 26% during the first year of the COVID-19 pandemic in 2020 [1]. Given this level of need, scaling up of quality and affordable mental health interventions and services is a key strategic action of the World Health Organisation special initiative for mental health [2].

Digital mental health interventions (DMHIs) are technology-based interventions that aim to prevent, educate, or treat mental health conditions, which are delivered via digital technologies such as mobile apps, internet websites, wearable devices, telephone, virtual reality and video games [3,4]. DMHIs can be self-guided, integrated with healthcare professional or peer support, in-person therapies, or offered through a combination of these approaches [5].

Given their low cost and resource constraints on in-person mental health services, DMHIs offer a scalable option for improving access to psychological support and therapies, potentially avoiding issues associated with stigma, as they can be personalised to individual needs [6,7]. Other potential benefits of DMHIs include averting delays or interruptions to support, expediting access to mental health advice and support as a complement to, or while waiting for, in-person services [8,9]. Furthermore, DMHIs have been suggested as a way of creating a tailored collection of different digital solutions—or a ‘poly-digital’ ecosystem—to address different aspects of an individual’s mental wellbeing [10].

Current estimates state that there are 227,500 health apps available in the United Kingdom (UK) [11]. There is evidence of DMHIs for impacting positively on outcomes for people living with depression, anxiety and stress [12–14] and specific populations such as children and young people [15,16], college students [12] and employed people engaging with work-based support [17]. A meta-analysis of 24 studies reporting on 7 of 48 DMHIs available via the National Health Service (NHS) in the UK for depression, anxiety or stress found small, but significant effects on outcomes, although attrition rates were high (31%) [18]. The authors concluded DMHIs are promising wait list interventions [18].

Given that only a small proportion of DMHIs have been subjected to an RCT evaluation [18], user preferences become critically important, and strategies are needed to empower them to make informed choices from the multitude of currently available DMHIs that are congruent with their preferences and values. Shared decision-making (SDM) is a person-centred approach whereby healthcare professionals and patients work collaboratively to identify and make an informed choice from the available options that is consistent with patient preferences and values [19]. There are few data on user preferences for features of DMHIs. Uptake of DMHIs will be highly dependent on whether they are perceived to be a good fit to people’s preferences, values and culture, and other preference and value sensitive features such as time commitment, anonymity, and cost [20,21].

SDM is frequently supported by a structured patient decision aid, which provides evidence-based information on the available options, benefits, risks/adverse events and likely outcomes. These tools impact positively on patients’ knowledge of available options, perception of risk, decisional conflict, and clarity about their preferences on available options, clinician–patient communication and active involvement of patients in decision making [22].

A decision aid for DMHIs does not currently exist. An important component of a decision aid in this context is clear information on features of DMHIs that are likely to be sensitive to individual preferences. An evidence based decision aid to support people experiencing mental health conditions would serve to engage them in SDM discussions with clinicians about whether to engage with a DMHI (indirectly addressing digital exclusion through better understanding of the digital offer), and facilitate identification of their preferred choice(s) of DMHI (as either the sole component of therapy, or as a precursor to prepare them to better engage with face-to-face delivery, leading to better outcomes for service users and their relatives).

### Aim

We aimed to develop a classification system of preference sensitive features of DMHIs to embed within a patient decision aid to support SDM.

### Objectives

1. Identify DMHIs that have been used (or available for use) in the UK
2. Classify the features of DMHIs that are likely to be sensitive to individual preferences
3. To develop questions to embed within a decision aid to elicit user preferences on features of DMHIs

## Review methods

### Search strategy

In order to develop a search strategy to identify DMHIs for inclusion in this review, a series of interactive meetings were held with a multi-disciplinary steering group of experts by experience (VN, SB, KD, KR), academics and practitioners from disciplines including mental health nursing (JB), health psychology (DF, LR), medical sociology (RW), occupational therapy (GB) and human computer interaction (TAB) and representatives from VCSE organisations providing mental health support and treatment (AC, LG). Electronic searching of bibliographic databases to identify DMHIs was ruled out in early discussions for being unable to address the aim and objectives of the review. A search of bibliographic databases would only identify DHMIs with published evaluations, and these DMHIs may no longer be available. Our aim was to classify the features of currently available DMHIs that have been used by adults or were available in the UK at the time of the review, as opposed to appraising the quality of the evidence-base for DMHIs.

The steering group identified DMHIs using the following methods:

1. A systematic review of DMHIs for depression, anxiety and stress available from the NHS in the UK [18]
2. Eight digitally enabled therapies to treat anxiety and depression conditionally recommended by the National Institute of Health and Care Excellence (NICE) in the UK [14].
3. DMHIs that people had used (or had been offered, but decided not to use) identified from responses to an online-survey (*n* = 46 respondents) and semi-structured interviews (*n* = 13 participants), which were conducted as part of the wider study to co-produce a decision aid for DMHIs [23].
4. The steering group compiled a list of DMHIs that were currently available based on their personal experience.

*The first two methods helped to identify DMHIs which have been recommended for use within the NHS in the UK and by NICE (the guideline producing organisation in the UK). Methods three and four aimed to identify additional DMHIs that were known to be available or used by mental health service users and direct care providers.*

### Review criteria

Eligible DMHIs had to meet the following criteria:

- Target population age ≥16 years
- Targeted a specific mental health condition(s) (e.g., depression, anxiety) or symptom (e.g., sleep)
- Delivered through any digital system such as internet websites, mobile apps, electronic messaging, video-conferencing (including virtual reality-based and wearable-integrated DMHIs) or through blended approaches of these systems”.
- Evidence of availability in the UK at the time of the review

### Data extraction

The data extraction form consisted of the four dimensional classification system developed by Gagnon and colleagues [24] consisting of ***System*** (for example, website, software, mobile app, electronic messaging); ***Function*** (decision support [screening or prompts/alerts], communication [one-way transmission, communication with healthcare providers or peer to peer], therapy [CBT, other psychotherapy, gamification] and monitoring [provider or self-monitoring]); ***Timing of Communication*** (DMHIs with synchronous or asynchronous communication with healthcare providers); and ***Facilitation*** (entirely or partially support by healthcare providers, self-guided)*.*

In addition, from discussions with the steering group, and findings from the online survey and interview study, we added further data fields: **Target population** (e.g., people living with depression); **Duration and recommendations for intensity** [frequency of engagement]; **Cost and access** [free to access, free to access via NHS referral or other referral, fee for a personal subscription); **Research evidence** (DMHI has evidence of effectiveness from at least one positive randomised controlled trial). The therapy sub-component of the classification system developed by Gagnon and colleagues [24] was amended to CBT, other psychological therapy, other psychotherapy or counselling approach, gamification.

The data extraction form was piloted with five DMHIs. Three reviewers (GB, DF, LR) independently extracted data on each DMHI using information on developer websites or other information available in the public domain. Published research for DMHIs included in the review was identified from searches of electronic databases (Medline, PsycINFO, and Google Scholar). A fourth reviewer (TAB) independently reviewed the data extraction for each DMHI, with any amendments resolved through discussion with the review team.

### Data synthesis

The resultant classification system was reviewed by the study authors (including people with lived experience of mental health conditions) to develop a series of questions to embed within a decision aid to facilitate the elicitation of user preferences and values on the features of DMHIs. This process was guided by guidelines on the development of decision aids [25–27], and a readability assessment available in MS Word (Flesch–Kincaid Grade Level [28]) to maximise accessibility to users with a range of reading abilities.

## Findings

**Fig 1:**
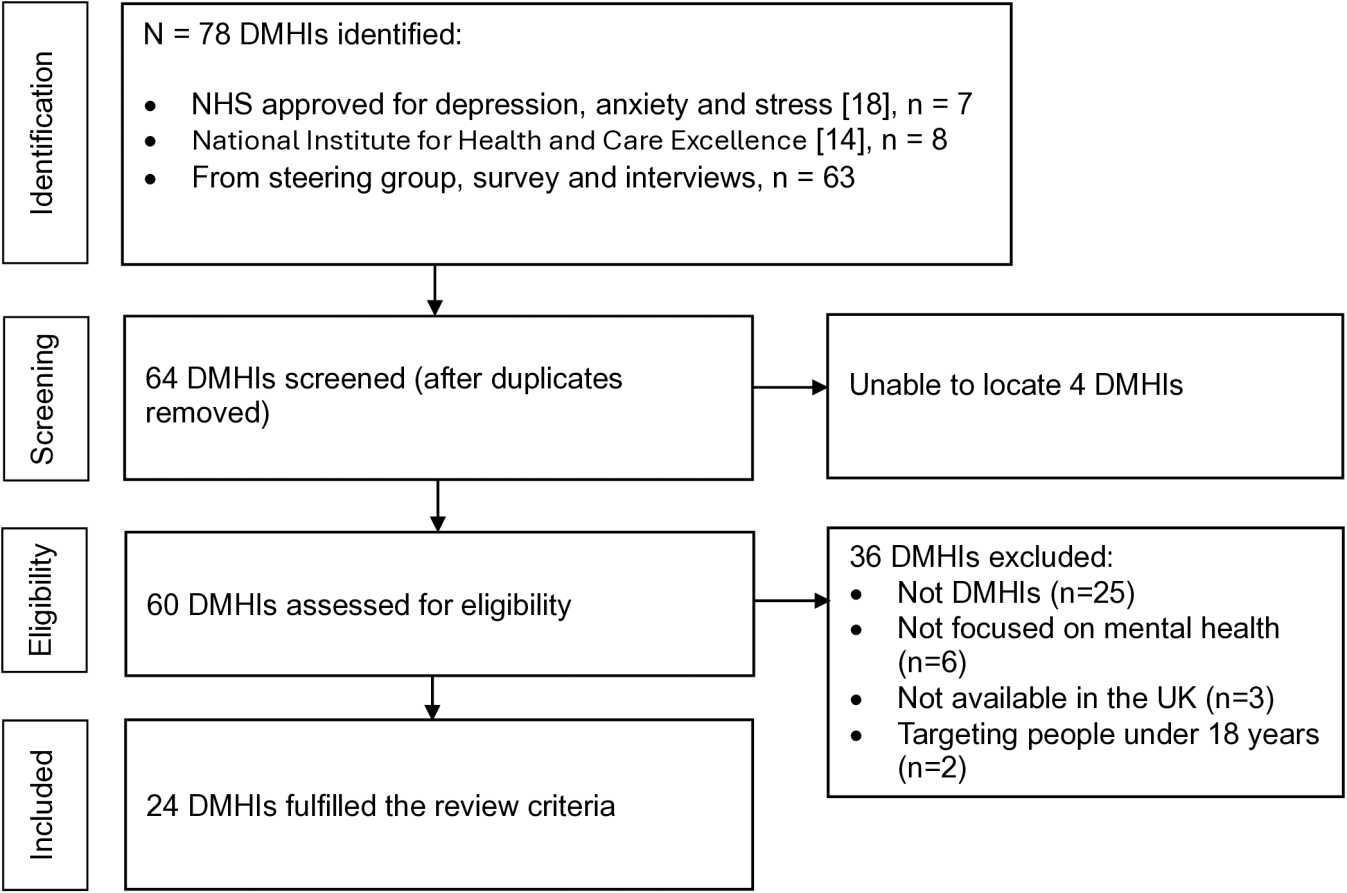
A flowchart of the process used to identify digital mental health interventions.

A total of 64 DMHIs were identified after removal of duplicates. Four DMHIs were excluded as their existence could not be verified. Out of the remaining 60 DMHIs, 36 were excluded with reasons, with 24 included in the review (Fig 1).

A summary of the features of the 24 DMHIs [29–52] included in the review is presented in Table 1.

**Table 1:** Summary of DMHIs included in the review.

DMHI	Target Population	System	Function(s)	Timing of communication, Duration and Intensity	Facilitation	Cost and Access
Beating the Blues [29]	People with mild to moderate depression and anxiety	Website	Therapy (CBT) Communication (one-way transmission of information)	Time - N/A 8 therapy sessions—50-min (one per week), plus homework sessions	Self-guided	If locally commissioned, free to access through NHS via professional referral Fee for a personal subscription
Moodgym [30]	People at risk, or living with anxiety and depression	Website	Therapy (CBT) Communication (one-way transmission of information)	Time—N/A Duration/intensity—not specified	Self-guided	If locally commissioned, free to access through NHS via professional referral Free with corporate subscription Fee for a personal subscription
IESO [31]	People experiencing anxiety/worry; depression; obsessive-compulsive disorder; post-traumatic stress disorder; phobias; sleep problems; social anxiety; stress	Electronic messaging	Therapy (CBT) Communication (with healthcare provider)	Asynchronous communication (electronic messaging) 30–60 min weekly live-typed therapy sessions	Entirely supported by healthcare professional	If locally commissioned, free to access through NHS with professional referral Fee for a personal subscription
Headspace [32]	People experiencing stress, poor sleep or wanting to find happiness/more time to relax	App	Therapy (Mindfulness) Communication (one-way transmission of information) Monitoring (no of days meditated and for how long)	Time—N/A Meditations last between 3 and 20 min, guided sleep sessions between 45 and 55 min. Suggested daily use of 10 and 45 min	Self-guided	Free App with in-App purchases or monthly subscription (discounts for students Corporate subscription Option for free trial
SilverCloud Health [33]	People living with depression, anxiety, depression and anxiety, insomnia and sleep issues, OCD, panic, phobias, ADHD.	Website and App Can include wearable devices	Therapy (CBT) Communication (one-way transmission of information; with healthcare professional) Monitoring Decision-support (prompts and alerts)	Asynchronous communication (messaging feature) and synchronous (telephone or video call) Recommends completing one module per week, each module taking 1 h to complete	Self-guided or partially supported by a healthcare professional	Free to access with referral (inc. NHS) from health insurers, employers, health professional, higher education institution
My Black Dog [34]	People living with mental health symptoms/conditions	Website (community focussed) Electronic messaging	Communication (one-way transmission of information, peer to peer)	Asynchronous communication (peer-to-peer messaging) Duration/intensity—not specified	Self-guided	Free to access for anyone in England and Wales
Inform Scotland (previously Moodjuice) [35]	People living with anorexia nervosa, bipolar disorder, anxiety, depression, bereavement, phobias	Website	Communication (one-way transmission of information)	Time—N/A Duration/intensity—not specified	Self-guided	Free to access
The Mindfulness App [36]	People who are struggling to sleep and experiencing stress	App	Therapy (mindfulness) Communication (one-way transmission of information) Decision-support (prompts and alerts to build or maintain meditation routine)	Some synchronous communication (content facilitated by coaches) 5-day introductory programme Beyond introductory programme, duration/intensity—not specified	Partially supported by a healthcare professional	Free App with basic features Payment option for premium app with enhanced content Free trial of premium
Meditation Oasis [37]	Anyone experiencing stress	App (also website and podcasts)	Therapy (meditation) Communication (one-way transmission of information)	Time—N/A Duration/intensity—not specified	Self-guided	Free App (basic features) Payment option for enhanced content
Qwell [38]	Adults (18^+^) across the UK requiring counselling support	Website, App, electronic messaging, podcasts	Therapy (counselling, meditation) Communication (one-way transmission of information, with healthcare professional, peer to peer)	Asynchronous communication (electronic messaging) Duration/intensity—not specified	Fully or partially supported by a healthcare professional	If locally commissioned, free to access through NHS via healthcare professional referral
Deprexis [39]	People with depression	Website, App	Therapy (CBT) Communication (one-way transmission of information) Monitoring (mood assessment) Decision-support (email/ text message support)	Time—N/A Ideally to be used 1–2 times per week for at least half an hour for each session	Self-guided	If locally commissioned, free to access through NHS via healthcare professional referral Individual subscription option (one-off payment for 90 day access)
Spring: Guided self-help for PTSD [40]	People with post-traumatic stress disorder of mild to moderate severity	Website, App	Therapy (CBT) Communication (one-way transmission of information, with healthcare professional) Monitoring (mood assessment) Decision-support (email/ text message support)	Synchronous communication (video calls) Duration/Intensity—not specified	Partially supported by a healthcare professional	If locally commissioned, free to access through NHS via healthcare professional referral
Calm [41]	People with sleep problems/ living with stress and anxiety	App	Therapy (based on CBT, ACT and DBT) Communication (one-way transmission of information)	Time—N/A Introductory programme—7 days Feature ‘daily calm’ takes 10 min Meditations last 2–60 min	Self-guided	Free App with basic features Payment option for premium app with enhanced content Option for corporate subscription
Mind [42]	Support for anyone with a mental health need (refers to a range of conditions, including depression and anxiety)	Website	Communication (one-way transmission of information, peer to peer)	Asynchronous communication (peer-to-peer messaging) Duration/intensity—not specified	Self-guided	Free to access
MoodTracker [43]	For people wanting to track health measurements such as mood, anxiety, sleep, water-intake, medication	Website, App, electronic messaging	Communication (peer to peer) Monitoring (reminders to take measurements)	Time—N/A Duration/intensity—not specified	Self-guided	Free App with basic features Payment required for enhanced content
Relax and Sleep Well [44]	People experiencing problems with sleep and meditation for anxiety	App	Therapy (hypnosis and meditation) Communication (one-way transmission of information)	Time—N/A Duration/intensity—not specified	Self-guided	Free App with basic features
Wysa [45]	People who are experiencing low mood, stress, anxiety or those interested in improving their emotional resilience	App, electronic messaging	Therapy (CBT, DBT, meditation) Communication (one-way transmission of information, with AI coach) Premium version (with healthcare professional)	Asynchronous communication (premium version—electronic messaging with health professional) Healthcare professional sessions last 30 min Messages outwith scheduled sessions responded ≤24 h	Fully supported by a healthcare professional	Free App with basic features Payment option for premium version with enhanced content
Betterhelp [46]	Anyone who wants to access mental health support	Website, App, electronic messaging	Therapy (counselling) Communication (with health professional)	Synchronous communication (live sessions with counsellor) Duration/intensity—not specified	Fully supported by a healthcare professional	Individual payment - £40-£70 per week Institution subscription model for employees
Finch: Self-care Pet [47]	Anyone who wants to focus on their own self-care	App	Therapy (gamification - self-care through taking care of a virtual pet) Communication (one-way transmission of information) Monitoring (mood checks, activity tracking	Time—N/A Duration/intensity—not specified	Self-guided	Free App with basic features (in-app purchases)
Every Mind Matters [48]	Anyone who wants support with their mental health	Website	Communication (one-way transmission of information)	Time—N/A Duration/intensity—not specified	Self-guided	Free webpages with signposts to free Apps
Stay Alive App [49]	For people experiencing suicidality or people who concerned about someone else	App	Communication (one-way transmission of information) Decision support (prompts to seek help) Storage (other function)	Time—N/A Duration/intensity—not specified	Self-guided	Free App
Decider Skills [50]	People engaged with mental health services/ anyone to manage their mental health (anxiety, low mood)	App (supported by website)	Therapy (CBT) Communication (one-way transmission of information)	Time—N/A Duration/intensity—not specified	Self-guided	Free App One-off payment for access to extra resources
Insight Timer [51]	People experiencing difficulty with sleep, anxiety or distress	App	Therapy (mindfulness and meditation) Communication (one-way transmission of information) Monitoring (tracking thoughts and feelings)	Time—N/A Duration/intensity—not specified	Self-guided	Free App Free basic corporate package Premium corporate package—price based on number of employees
Pzizz [52]	People struggling with sleep	App	Therapy (music-based relaxation) Communication (one-way transmission of information)	Time—N/A Can set personal listening time for up to 24 h	Self-guided	Free App (basic features) Payment option for enhanced content

### Target population

Thirteen (54%) DMHIs made specific reference to people living with low mood [45,50], depression [29–31,33,35,39,42] or anxiety [29–31,33,35,41–45,50,51]. People experiencing ‘stress’ was mentioned by four DMHIs [32,36,37,41]. DMHIs also targeted people living post-traumatic stress disorder [31,40], obsessive-compulsive disorder [31], anorexia nervosa [35], bi-polar disorder [35], phobias [31,35] and suicidality [49].

Eight DMHIs also referred to anyone who wanted support with their mental health [32,34,38,42,45–48].

### System

Eight DMHIs were delivered entirely [32,36,41,44,47,49,51,52] through a mobile app, with a further eight using Apps in combination with websites [33,37–40,43,46,50] and four used Apps in combination with electronic messaging [38,43,45,46]. Five were exclusively delivered through websites [29,30,35,42,48], with one using a combination of website and electronic messaging [34]. One DMHI was exclusively delivered through electronic messaging [31]. Three were delivered using a combination of apps, websites and electronic messaging [38,43,46]. Two DMHIs also used podcasts [37,38].

### Function

Eighteen (75%) DMHIs had a function of therapy [29–33,36–41,44–47,50–52]. The most frequently cited therapeutic model in these DMHIs was Cognitive Behavioural Therapy (CBT), which was offered by nine DMHIs [29–31,33,39–41,45,50]. Two DMHIs offered Dialectical Behavioural Therapy (DBT) [41,45]. One offered Acceptance and Commitment Therapy (ACT) as well as CBT and DBT [41]. Three DMHIs offered mindfulness [32,36,51] and four others offered meditation [37,44,45,51]. Counselling was provided by two DMHIs [38,46]. Three DHMIs offered additional therapeutic methods - gamification in the form of encouraging people to attend to own self care through taking care of a virtual pet [47], music-based relaxation [52] and hypnosis [44].

Twenty one (88%) DMHIs had one-way transmission of information [29,30,32–42,44,45,47–52]. Ten also had two-way communication with a healthcare provider [31,33,38,45,46], peer to peer communication [34,38,42,43] or with an AI-coach [45].

Seven DMHIs had a function of monitoring [32,33,39,40,43,47,51], for example mood or time spent meditating. Five DMHIs had decision-support functions [33,36,39,40,49] with features such as prompts and alerts to build or maintain meditation routine [36] or prompts to seek help when experiencing suicidality [49]. One DMHI enabled a personalised storage function of items such as photos to support emotional regulation [49].

### Facilitation

Eighteen (75%) DMHIs were capable of being self-guided [20,29,32,34,35,37,39,41–44,47–52]. Seven were partially or entirely supported by healthcare professionals [31,33,36,38,40,45,46]. One was also guided by an AI coach [45].

### Timing of communication, duration and intensity

Only four DMHIs provided synchronous communication [33,36,40,46] and six provided asynchronous communication [31,33,34,38,42,45]. One offered both asynchronous and synchronous communication [33]. The remaining DMHIs were completely self-guided with no communication feature.

Six (25%) DMHIs stated or provided recommendations on duration and intensity of use [29,31–33,39,41]. One provided information on only duration of sessions [45]. One relaxation-based App [52] stated that users could personalise the duration and intensity.

### Cost and access

Eleven DMHIs could be accessed at no cost via a referral from the NHS in the UK [29–31,33,38–40], which varied based on geographical location/local commissioning arrangements, or access via a corporate subscription [30–33,41,46,51]. Six could be accessed outwith the NHS or corporate setting at no cost [34,35,42,48,49,51], although one was restricted to residents of England and Wales [34]. Ten were also available at no cost, but with access only to basic features, with a fee to gain access to premium content [32,36,37,41,43–45,47,50,52], with two of these offering a free trial [32,36]. Five offered access via personal subscriptions for a fee [29–31,39,46].

### Research evidence

Eight [29,30,32,33,36,39,41,51] of the 24 DMHIs included in the review had evidence of effectiveness from randomised controlled trials (Table 2)

**Table 2:** Summary of key findings of RCTs for DMHIs.

DMHI	Key findings
Beating the Blues [29]	Compared with usual treatment, participants assigned to Beating the Blues reported improved depression, negative attributional style, work and social adjustment, including treatment satisfaction. Participants with anxiety and positive attributional style, those with more severe anxiety improved more than those with less severe symptoms [53]
Moodgym [30]	Meta-analysis of 11 comparative studies (including RCTs) found effectiveness for depression symptoms, with a small effect size. Comparisons from six studies demonstrated MoodGYM’s effectiveness for improving anxiety symptoms (medium effect size) [54]
Headspace [32]	Across 14 randomised controlled trials of Headspace, depression improved in 75% of studies that evaluated it as an outcome. Findings were mixed for mindfulness, well-being, stress, and anxiety, but at least 40% of studies showed improvement for each of these outcomes [55]
SilverCloud Health [33]	72 participants (breast cancer survivors) were randomised to a 7-module guided online CBT intervention or treatment-as-usual (TAU) had lower Hospital Anxiety and Depression Scale total score scores than TAU at 2-months post-intervention [56] 361 participants were randomised (online CBT, 241; waiting-list, 120). Intention-to-treat analyses showed significant interaction effects for the PHQ-9 (*depression)* and GAD-7 (*anxiety*) in favour of iCBT at 8-weeks (further improvements were observed up to 12-months) [57]
The Mindfulness App [36]	81 participants were randomised to behavioural activation (BA) or mindfulness. The two interventions did not differ significantly in terms of PHQ-9 or BDI-II scores. For participants with higher severity of depression, BA was superior to mindfulness. Conversely, participants with lower initial severity, mindfulness was superior to BA [58]
Deprexis [39]	Meta-analysis of 12 deprexis-specific RCTs with a total of N = 2901 participants confirmed the effectiveness of deprexis for depression reduction at post-intervention (over 8-12 weeks across a broad range of initial symptom severity) [59] Compared participants allocated to the intervention condition (treatment as usual plus immediate access to Deprexis for 90 days, *n* = 94) with a control condition (treatment as usual and delayed access to Deprexis, after 8 weeks, *n* = 95). Deprexis improved significantly more than participants assigned to the delayed access control group on depression (PHQ–9) and general psychological state (Clinical Outcome in Routine Evaluation–Outcome Measurement). 21% and 7% of the participants in the Deprexis versus the control group respectively achieved remission compared with in the control group [60]
Calm [41]	In a sample of 88 participants (college students) there were significant differences in stress, mindfulness, and self-compassion between the intervention and control groups after adjustment for covariates post-intervention [61]
Insight Timer [51]	Anxiety (GAD-7) decreased more in the intervention (Insight Timer) than the control group (*n* = 28). Well-being (World Health Organization Well-Being Index -5) scores were improved in both the control and intervention groups, but was higher in the intervention group [62]

### Preference-elicitation statements for inclusion in a decision aid

Discussions with the multi-disciplinary steering group, with reference to the classification system and summary of research evidence for DMHIs was used to develop statements to elicit user preferences on features of DMHIs (Table 3). Timing of Communication (DMHIs with synchronous or asynchronous communication with healthcare providers) and Facilitation (entirely or partially support by healthcare providers, self-guided) were combined into one category. The steering group considered that people will have specific preferences for self-completion [not involving communication with others] and communication with a healthcare professional either in real-time [via phone] or not [via email], with the latter, by definition, facilitated entirely or partially supported by healthcare providers. Therefore, a separate category of facilitation might potentially confuse potential users. Peer support was also added as a sub-category to Function.

**Table 3:** Classification system and questions to elicit preferences on features of DMHIs.

Feature	Sub-category	Statements to elicit preferences	Notes
Target Population	Specific diagnosis or symptoms	I would prefer a DMHI designed for: (a) People diagnosed with a specific mental health condition (e.g., depression and/or anxiety) (b) People who are experiencing symptoms of a mental health condition, or would like support with their mental health	**IF (a)** Depression Anxiety Depression and anxiety Suicidal thoughts PTSD etc
Function	Therapy Decision support Monitoring Peer Support	I would prefer a DMHI to provide: (a) A talking treatment (Therapy)* (b) Prompts and alerts to help manage my mental health (c) Ways to keep track of my mental health (d) Peer support (people who have lived experience of mental health conditions)	If only (b) or (c) chosen, then go to SYSTEM
	Therapy*	I would prefer a talking treatment (therapy) based on: (a) Cognitive Behavioural Therapy (b) Dialectical Behaviour Therapy (c) Counselling (d) Meditation or mindfulness (e) The type of therapy is not important to me	
Timing of Communication and Facilitation	Synchronous Asynchronous Supported by healthcare provider Chat-bot Self-guided	I would prefer a DMHI that: (a) Involves communication/support from a health professional in real-time/ or via email (b) Uses a conversational agent (also known as a chatbot) (c) I can work through it myself (self-guided)	
System	Mobile App Internet/ website Telehealth Electronic messaging	I would prefer a DMHI that uses: (a) An App on a phone or tablet (e.g., Ipad) (b) Website (via a desktop PC, phone or tablet). (c) Video-conferencing (e.g., Zoom) (d) Electronic messaging	
Cost and Access	Free (referral) Free for basic features (costs for premium version) One-off payment or regular subscription	I would prefer a DMHI that is: (a) Free to access via referral by the NHS or my workplace (b) Free to access myself, but may involve a fee to access the premium version (c) Free to access myself to the full version (d) A fee to gain access myself (one-off payment/ subscription)	
Research Evidence	Evidence of • Effectiveness (meta-analysis or RCT) • Acceptability or satisfaction	I would prefer a DMHI with: (a) Strong research evidence for benefits to mental health (b) Evidence that people living with mental health conditions are satisfied with the DMHI (c) This is not important to me	

The statements to elicit preferences had a Flesch-Kincaid Grade Level of 8.

**Fig 2:**
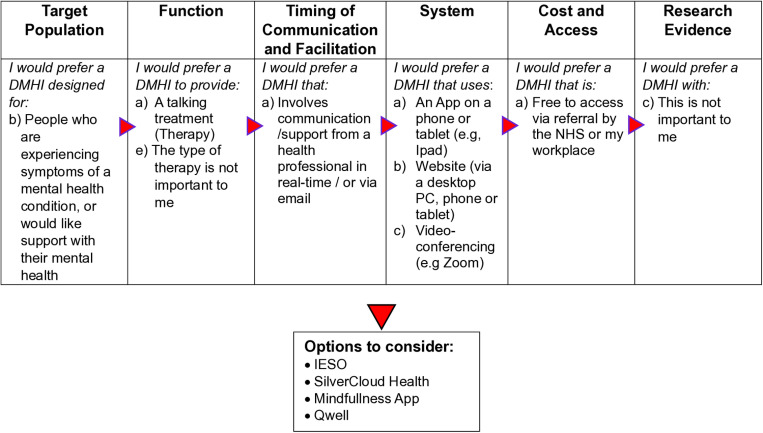
Example of how the preference elicitation statements can help people to identify options for DMHIs.

The decision aid will help people to identify potential options of DMHIs based on their preferences and discuss these options with a supporter or health professional. We provide an illustrative example in Fig 2.

## Discussion

We used a pragmatic approach to identify 24 DMHIs with evidence of current use or availability in the UK, which varied across seven key features that are likely to be preference-sensitive (target population, function, time and facilitation, system, cost and access, and research evidence). The heterogeneity of features of DMHIs in this review (and available more widely) represents a considerable challenge for users to identify a DMHI that meets their needs and is consistent with their preferences. It is likely that more than one DMHI is needed to address the full range of user needs, which reflects the concept of ‘poly-digital’, where many different features across multiple DMHIs (e.g., one for therapy, and others for mood monitoring and mindfulness) can each address different facets of wellbeing needs, potentially resulting in an aggregation of marginal gains [10].

Therefore, accessible summaries of features of DMHIs are imperative for users and healthcare providers to engage in SDM discussions to identify the best option(s) for DHMIs to optimising uptake and subsequent outcomes. Engaging people in SDM discussions about DMHIs may help to reduce dropout rates, which are high compared with in person therapy [63], and address ‘App Fatigue’ arising from difficulty in navigating the huge number of available options [11]. A traditional decision aid, where features for all the available options are presented, along with probabilities for the range of outcomes is not feasible for DHMIs. Furthermore, relatively few DMHIs are supported by evidence from randomised controlled trials, and where such evidence does exist, there are issues associated with conflicts of interest. For example, a review of the evidence for Headspace identified that 50% (7/14) of RCTs reported a conflict of interest that involved the owner of the DMHI [55].

Therefore, a decision aid for DMHIs would benefit from focussing on features of DMHIs using the classification system developed in this review. Identified user preferences for features of DMHIs arising from a SDM interaction would then inform a discussion about available options that are linked to an electronic repository of DMHIs (for example, Beacon: https://beacon.anu.edu.au/) [64] using our classification system.

Seven DMHIs in our review were facilitated (at least in part) by health professionals and for others, health professionals will have variable involvement in supporting people to consider using DMHIs. Therefore, further proliferation of DMHIs would necessitate rapid workforce development across sectors involved in providing mental health support [65]. Many healthcare providers (within and outwith mental health contexts) may not have received training on the use of DMHIs in their practice and core competencies such as evidence, integration, security and privacy, ethics, and cultural considerations are recommended [66]. It is essential that SDM discussions about DMHIs, including within a decision aid to support such discussions includes an overview on data privacy, security, and ethical considerations to maximise both user safety and trust in these types of interventions.

### Limitations

The DMHIs included in our review do not represent the entirety of currently available DMHIs in the UK, or globally in particular emerging systems such as VR-based and wearables. In a paper published in 2020, it was estimated that there are between 10,000 and 22,750 mental health apps [66]. The field of digital mental health is constantly in flux, with new and updated versions of DHMIs being released. Given this ‘temporal validity’, it is likely that several DMHIs in this review will no longer be available or have since been updated with additional features. We did not identify any DMHIs based on other emerging technologies, such as VR-based or wearable-integrated DHMIs, which are increasingly being applied in mental health contexts [67]. It is also possible that information about features of DMHIs may exist that is not explicitly stated on developer websites or other information available in the public domain.

In order to identify DMHIs for inclusion in this review, we drew on a multi-disciplinary steering group (experts by experience, academics, and representatives from VCSE organisations providing mental health support and treatment), data from an online survey and semi-structured interviews, plus relevant literature. This represented a range of perspectives and experiences to identify DMHIs, although we acknowledge that members of the steering group, and respondents to the survey and interviews, were primarily located around one region of the UK (north east). Future research on the classification system would benefit from wider engagement with service users and direct care providers across the UK and other countries to balance these perspectives.

A precursor to making preference-based choice of DMHIs, is establishing a person’s preference for digital technology, and addressing digital literacy and access to digital resources. Therefore, a decision aid would also benefit from initially establishing a person’s preference for use of DMHIs with clear and unbiased information on their potential benefits and challenges. If this supported discussion yields a positive preference, options to support digital literacy and access to an individual’s preferred DMHIs could then be provided.

## Conclusions

Our review has identified DMHIs with evidence of use or availability in the UK at the time of the review. The statements designed to elicit user preferences on features of DHMIs have been embedded into a prototype decision aid. Future work will involve subjecting our decision aid for DMHIs to acceptability and usability testing, alongside the development of a digital explainer to convey clear information on DMHIs and the importance of engaging in a SDM process to identify the best option(s) for the individual that matches their needs and preferences for features of DMHIs.

## Supporting information

S1 Text Glossary of terms (DOCX)

## References

[1] World Health Organisation. World mental health report: transforming mental health for all. 2022. Available from: https://iris.who.int/bitstream/handle/10665/356119/9789240049338-eng.pdf?sequence=1

[2] World Health Organisation. The WHO special initiative for mental health (2019-2023). Universal Health Coverage for Mental Health; 2017. Available from: https://iris.who.int/bitstream/handle/10665/310981/WHO-MSD-19.1-eng.pdf?sequence=1&isAllowed=y

[3] Mohr DC, Burns MN, Schueller SM, Clarke G, Klinkman M. Behavioral intervention technologies: evidence review and recommendations for future research in mental health. Gen Hosp Psychiatry. 2013;35(4):332–8. doi: 10.1016/j.genhosppsych.2013.03.008 23664503 PMC3719158

[4] Park SY, Nicksic Sigmon C, Boeldt D. A framework for the implementation of digital mental health interventions: the importance of feasibility and acceptability research. Cureus. 2022;14(9):e29329. doi: 10.7759/cureus.29329 36277565 PMC9580609

[5] Muñoz RF. The efficiency model of support and the creation of digital apothecaries. Clin Psychol Sci Pract. 2016;24(1):46–9.

[6] Himle JA, Weaver A, Zhang A, Xiang X. Digital mental health interventions for depression. Cogn Beha Pract. 2022;29(1):50–9. doi: 10.1016/j.cbpra.2020.12.009

[7] Taylor CB, Graham AK, Flatt RE, Waldherr K, Fitzsimmons-Craft EE. Current state of scientific evidence on Internet-based interventions for the treatment of depression, anxiety, eating disorders and substance abuse: an overview of systematic reviews and meta-analyses. Eur J Public Health. 2021;31(31 Suppl 1):i3–10. doi: 10.1093/eurpub/ckz208 32918448 PMC8495688

[8] Philippe TJ, Sikder N, Jackson A, Koblanski ME, Liow E, Pilarinos A, et al. Digital health interventions for delivery of mental health care: systematic and comprehensive meta-review. JMIR Ment Health. 2022;9(5):e35159. doi: 10.2196/35159 35551058 PMC9109782

[9] Baños RM, Herrero R, Vara MD. What is the current and future status of digital mental health interventions?. Span J Psychol. 2022;25:e5. doi: 10.1017/SJP.2022.2 35105398

[10] Bond RR, Mulvenna MD, Potts C, O’Neill S, Ennis E, Torous J. Digital transformation of mental health services. Npj Ment Health Res. 2023;2(1):13. doi: 10.1038/s44184-023-00033-y 38609479 PMC10955947

[11] Healthcare Communications UK. Striking a balance: navigating healthcare engagement in the age of app fatigue. Available from: https://healthcare-communications.com/striking-a-balance-navigating-healthcare-engagement-in-the-age-of-app-fatigue/#:~:text=There%20are%20currently%20a%20massive,downloaded%20more%20than%2010m%20times

[12] Lattie EG, Adkins EC, Winquist N, Stiles-Shields C, Wafford QE, Graham AK. Digital mental health interventions for depression, anxiety, and enhancement of psychological well-being among college students: systematic review. J Med Internet Res. 2019;21(7):e12869. doi: 10.2196/12869 31333198 PMC6681642

[13] Martinengo L, Stona A-C, Griva K, Dazzan P, Pariante CM, von Wangenheim F, et al. Self-guided cognitive behavioral therapy apps for depression: systematic assessment of features, functionality, and congruence with evidence. J Med Internet Res. 2021;23(7):e27619. doi: 10.2196/27619 34328431 PMC8367167

[14] National Institute for Health and Care Excellence. Eight digitally enabled therapies to treat depression and anxiety in adults conditionally recommended by NICE. Available from: https://www.nice.org.uk/news/article/eight-digitally-enabled-therapies-to-treat-depression-and-anxiety-in-adults-conditionally-recommended-by-nice.

[15] Lehtimaki S, Martic J, Wahl B, Foster KT, Schwalbe N. Evidence on digital mental health interventions for adolescents and young people: systematic overview. JMIR Ment Health. 2021;8(4):e25847. doi: 10.2196/25847 33913817 PMC8120421

[16] Fried R, DiSalvo M, Farrell A, Biederman J. Using a digital meditation application to mitigate anxiety and sleep problems in children with ADHD. J Atten Disord. 2022;26(7):1033–9. doi: 10.1177/10870547211025616 34865550

[17] Carolan S, Harris PR, Cavanagh K. Improving employee well-being and effectiveness: systematic review and meta-analysis of web-based psychological interventions delivered in the workplace. J Med Internet Res. 2017;19(7):e271. doi: 10.2196/jmir.7583 28747293 PMC5550734

[18] Simmonds-Buckley M, Bennion MR, Kellett S, Millings A, Hardy GE, Moore RK. Acceptability and effectiveness of NHS-recommended e-therapies for depression, anxiety, and stress: meta-analysis. J Med Internet Res. 2020;22(10):e17049. doi: 10.2196/17049 33112238 PMC7657731

[19] Hargraves I, LeBlanc A, Shah ND, Montori VM. Shared decision making: the need for patient-clinician conversation, not just information. Health Aff (Millwood). 2016;35(4):627–9. doi: 10.1377/hlthaff.2015.1354 27044962

[20] Borghouts J, Eikey E, Mark G, De Leon C, Schueller SM, Schneider M, et al. Barriers to and facilitators of user engagement with digital mental health interventions: systematic review. J Med Internet Res. 2021;23(3):e24387. doi: 10.2196/24387 33759801 PMC8074985

[21] Fisher A, Corrigan E, Cross S, Ryan K, Staples L, Tan R, et al. Decision-making about uptake and engagement among digital mental health service users: a qualitative exploration of therapist perspectives. Clin. Psychol. 2023;27(2):171–85. doi: 10.1080/13284207.2022.2163157

[22] Stacey D, Lewis KB, Smith M, Carley M, Volk R, Douglas EE, et al. Decision aids for people facing health treatment or screening decisions. Cochrane Database Syst Rev. 2024;1(1):CD001431. doi: 10.1002/14651858.CD001431.pub6 38284415 PMC10823577

[23] Rehackova L, Bradley G, Nunn V, Devereaux K, Barker J, Burrows S, et al. Co-production of a decision aid to facilitate shared decision-making about technology-assisted mental health support. 38th Annual Conference of the European Health Psychology Society. Health Psychology for a Sustainable Future. 3–6 September, 2024. Cascais, Portugal.

[24] Gagnon M-P, Sasseville M, Leblanc A. Classification of digital mental health interventions: a rapid review and framework proposal. Stud Health Technol Inform. 2022;294:629–33. doi: 10.3233/SHTI220545 35612165

[25] Witteman HO, Maki KG, Vaisson G, Finderup J, Lewis KB, Dahl Steffensen K, et al. Systematic development of patient decision aids: an update from the IPDAS collaboration. Med Decis Making. 2021;41(7):736–54. doi: 10.1177/0272989X211014163 34148384 PMC8664088

[26] Witteman HO, Ndjaboue R, Vaisson G, Dansokho SC, Arnold B, Bridges JFP, et al. Clarifying values: an updated and expanded systematic review and meta-analysis. Med Decis Making. 2021;41(7):801–20. doi: 10.1177/0272989X211037946 34565196 PMC8482297

[27] O’Connor A, Llewellyn-Thomas H, Dolan J, Kupperman M, Willis C. Section D: clarifying and expressing values. In: O’Connor A, Llewellyn-Thomas H, Stacey D, eds. IPDAS collaboration background document. International Patient Decision Aids Standards (IPDAS) Collaboration; 2005. p. 17–23. Available from: http://ipdas.ohri.ca/IPDAS_Background.pdf

[28] Microsoft Corporation. Get your document’s readability and level statistics. Available from: https://support.microsoft.com/en-gb/office/get-your-document-s-readability-and-level-statistics-85b4969e-e80a-4777-8dd3-f7fc3c8b3fd2

[29] Beating the Blues. No date. Available online at: https://www.maximusuk.co.uk/beating-the-blues

[30] Moodgym. No date. Available from: https://www.moodgym.com.au/.

[31] IESO. No date. Available from: https://www.iesohealth.com/.

[32] Headspace Inc. Headspace v3.313.0. [Mobile App]. 2024.

[33] Silvercloud Health. 2024. Available online at: https://www.silvercloudhealth.com/.

[34] My Black Dog. No date. Available online at: https://www.myblackdog.co/.

[35] Inform Scotland. 2023. Available online at: https://www.nhsinform.scot/illnesses-and-conditions/mental-health

[36] Reflectly ApS. The mindfulness app v5.44.0 [Mobile App]. 2024.

[37] Meditation Oasis. Meditation Oasis v7.4 [Mobile App]. 2022.

[38] Qwell. 2024. Available from: https://www.qwell.io/

[39] Deprexis. No date. Available from: https://deprexis.com/

[40] NHS Wales. ‘Spring: Guided self help for PTSD’. No date. Available from: https://traumaticstress.nhs.wales/events/tsw-conference-presentations-2022/guided-self-help-for-ptsd-spring/

[41] Calm.com. Calm v6.42.3 [Mobile App]. 2024.

[42] Mind. Available from: https://www.mind.org.uk/. 2024.

[43] MoodTracker. 2024. Available from: https://www.moodtracker.com/.

[44] Divinti Publishing Ltd. Relax and Sleep Well v8.7 [Mobile App]. 2024.

[45] Touchkin. Wysa v6.6.9 [Mobile App]. 2024.

[46] Betterhelp. 2024. Available from: https://www.betterhelp.com/.

[47] Finch Care Public Benefit Corporation. Finch: Self Care Pet v3.69.8 [Mobile App]. 2024.

[48] NHS. Every Mind Matters. No date. Available from: https://www.nhs.uk/every-mind-matters/.

[49] Grassroots Suicide Prevention. Stay Alive v3.29.3 [Mobile App]. 2024.

[50] Yuri Alves. The Decider Skills v1.2 [Mobile App]. 2023

[51] Insight Network Inc. Insight Timer v18.1.0 [Mobile App]. 2024

[52] Pzizz. Pzizz v5.0.38 [Mobile App]. 2024

[53] Proudfoot J, Ryden C, Everitt B, Shapiro DA, Goldberg D, Mann A, et al. Clinical efficacy of computerised cognitive-behavioural therapy for anxiety and depression in primary care: randomised controlled trial. Br J Psychiatry. 2004;185:46–54. doi: 10.1192/bjp.185.1.46 15231555

[54] Twomey C, O’Reilly G. Effectiveness of a freely available computerised cognitive behavioural therapy programme (MoodGYM) for depression: meta-analysis. Aust N Z J Psychiatry. 2017;51(3):260–9. doi: 10.1177/0004867416656258 27384752

[55] O’Daffer A, Colt SF, Wasil AR, Lau N. Efficacy and conflicts of interest in randomized controlled trials evaluating headspace and calm apps: systematic review. JMIR Ment Health. 2022;9(9):e40924. doi: 10.2196/40924 36125880 PMC9533203

[56] Akkol-Solakoglu S, Hevey D. Internet-delivered cognitive behavioural therapy for depression and anxiety in breast cancer survivors: results from a randomised controlled trial. Psychooncology. 2023;32(3):446–56. doi: 10.1002/pon.6097 36635249

[57] Richards D, Enrique A, Eilert N, Franklin M, Palacios J, Duffy D, et al. A pragmatic randomized waitlist-controlled effectiveness and cost-effectiveness trial of digital interventions for depression and anxiety. NPJ Digit Med. 2020;3:85. doi: 10.1038/s41746-020-0293-8 32566763 PMC7295750

[58] Ly KH, Trüschel A, Jarl L, Magnusson S, Windahl T, Johansson R, et al. Behavioural activation versus mindfulness-based guided self-help treatment administered through a smartphone application: a randomised controlled trial. BMJ Open. 2014;4(1):e003440. doi: 10.1136/bmjopen-2013-003440 24413342 PMC3902198

[59] Twomey C, O’Reilly G, Bültmann O, Meyer B. Effectiveness of a tailored, integrative Internet intervention (deprexis) for depression: updated meta-analysis. PLoS One. 2020;15(1):e0228100. doi: 10.1371/journal.pone.0228100 31999743 PMC6992171

[60] Lopes RT, da Rocha GC, Svacina MA, Meyer B, Šipka D, Berger T. Effectiveness of an internet-based self-guided program to treat depression in a sample of Brazilian users: randomized controlled trial. JMIR Form Res. 2023;7:e46326. doi: 10.2196/46326 37590052 PMC10472176

[61] Huberty J, Green J, Glissmann C, Larkey L, Puzia M, Lee C. Efficacy of the mindfulness meditation mobile app “calm” to reduce stress among college students: randomized controlled trial. JMIR Mhealth Uhealth. 2019;7(6):e14273. doi: 10.2196/14273 31237569 PMC6614998

[62] O’Donnell K, Dunbar M, Speelman D. Effectiveness of daily mindfulness meditation app usage to reduce anxiety and improve well-being during the COVID-19 pandemic: a randomized controlled trial. Cureus. 2023;15(7):e42432. doi: 10.7759/cureus.42432 37637657 PMC10448000

[63] Bayliss P, Willis J. An investigation of clients who drop out of the computerised cognitive behavioural therapy programme ‘Beating the Blues’. bpscpf. 2010;1(206):19–23. doi: 10.53841/bpscpf.2010.1.206.19

[64] Christensen H, Murray K, Calear AL, Bennett K, Bennett A, Griffiths KM. Beacon: a web portal to high-quality mental health websites for use by health professionals and the public. Med J Aust. 2010;192(S11):S40-4. doi: 10.5694/j.1326-5377.2010.tb03692.x 20528708

[65] Buck B, Kopelovich SL, Tauscher JS, Chwastiak L, Ben-Zeev D. Developing the workforce of the digital future: leveraging technology to train community-based mobile mental health specialists. J Technol Behav Sci. 2022:1–7. doi: 10.1007/s41347-022-00270-6 35967965 PMC9362666

[66] Schueller SM, Armstrong CM, Neary M, Ciulla RP. An Introduction to core competencies for the use of mobile apps in cognitive and behavioral practice. Cogn Behav Pract. 2022;29(1):69–80. doi: 10.1016/j.cbpra.2020.11.002

[67] Jerdan SW, Grindle M, van Woerden HC, Kamel Boulos MN. Head-mounted virtual reality and mental health: critical review of current research. JMIR Serious Games. 2018;6(3):e14. doi: 10.2196/games.9226 29980500 PMC6054705

