# Supplementary material for: Classifying the features of digital mental health interventions to inform the development of a patient decision aid
Source: PLOS Digit Health. 2025 Mar 26;4(3):e0000752. doi: 10.1371/journal.pdig.0000752 (PMC11942417; doi:10.1371/journal.pdig.0000752)
Supplement: S1 Text — (DOCX) [file pdig.0000752.s001.docx]

**Classifying the Features of Digital Mental Health Interventions to Inform the Development of a Patient Decision Aid**

**Glossary of technical terms**

| Acceptance and Commitment Therapy (ACT) | A therapeutic approach which focuses on accepting thoughts and feelings and work towards goals. |
| --- | --- |
| Access | The means by which a person gets an opportunity to use a digital system. |
| Application or App | A self-contained software package that allows users to perform specific tasks on an electronic device. |
| Artificial Intelligence or AI | The simulation of human intelligence using machines. |
| Classification system | A way of grouping and organising data |
| Cognitive Behavioural Therapy (CBT) | A therapeutic approach which focuses on helping people to understand and change the way they think and behave |
| Cost | The amount that must be paid by the user, before or during the use of a digital system. |
| Decision-support | Use of digital systems to help people to make decisions. |
| Dialectical Behavioural Therapy (DBT) | A specific therapeutic approach, based on CBT, but focuses more specifically on the emotional and social aspects of daily life. |
| Digital Mental Health Intervention (DMHI) | Technology-based interventions that aim to prevent or treat mental health conditions or educate people about their mental health. |
| Duration | The length of time needed or recommended to engage with a digital system |
| Electronic messaging | Information transmitted in digital form between systems. It can include text messages, emails and instant messaging. |
| Facilitation | How engagement with the digital system is supported (either fully or partially) by professionals, peers or is self-guided. |
| Function | The operations that are expected of a system. The functions of a digital system could include communication, monitoring, supporting decisions or facilitating therapy. |
| Gamification | The application of elements of game playing to other areas of daily life, for example learning about or managing mental health. |
| Intensity | The amount, dose or level of engagement with a digital system. |
| Monitoring | The use of a digital system to maintain surveillance. It often involves record keeping to enable feedback. |
| Peer support | People with similar experiences who come together to provide support |
| Podcast | A digital audio file, or series of audio files, listened to on a digital device. |
| Prototype | A first or preliminary version of a product, often built to test a concept or process. |
| Psychotherapy | An umbrella term for a variety of approaches that help people to identify and work through a range of emotions, thoughts and behaviours. Sometimes called talking therapy. |
| Self-guided | Guided and controlled by oneself. Sometimes called self-directed. |
| Shared decision aid | Tools which are designed to support shared decision-making between people and professionals. They provide evidence-based information about treatment, care and support options on a particular health or care topic. Sometimes called decision support tools. |
| Software | A set of instructions, data or programs used to operate computers. It is the opposite of hardware which describes the physical aspects of a computer. |
| System | A type of electronic system that can store, use and communicate digital information. Examples include websites, applications, electronic messaging and social media. |
| Target population | A group of individuals that a product or intervention is aimed towards. |
| Telehealth | The use of communication technologies to provide health care at a distance. |
| Timing of communication | The requirement for engagement with a system at a set time, at the same time as others (synchronous) or at a time independent of others (asynchronous) |
| Video game | A game that is played electronically and requires interaction with a device such as a joystick, keyboard or touchscreen. |
| Virtual reality | Computer-generated environment with scenes and objects that appear real, can be interacted with, and enable the user to feel immersed. |
| Website | A collection of files, accessed through a web address, typically produced by a single organisation |
| Wearable device | An electronic device designed to be worn on a user’s body. Can include watches, jewellery, glasses and headsets. |
